# Acceptability and applicability of using virtual reality for training mass casualty incidents- a mixed method study

**DOI:** 10.1186/s12909-025-07319-z

**Published:** 2025-05-19

**Authors:** Sara Heldring, Maria Jirwe, Jonas Wihlborg, Veronica Lindström

**Affiliations:** 1 Department of Health Promoting Science, Sophiahemmet University, Lindstedtsvägen 8, SE-114 86, Box 5605, Stockholm, Sweden; 2 AISAB, Ambulance Service in Region of Stockholm, Stockholm, Sweden; 3 Department of Health Sciences, Swedish Red Cross University, Box 1059, 141 21 Stockholm, Huddinge Sweden; 4 School of Health and Welfare, Dalarna University, 791 88 Falun, Sweden; 5 Department of Nursing, Section of Ambulance Service, Umeå University, 901 87 Umeå, Sweden

**Keywords:** Critical Realism, First Responders, Mass Casualty Incident, Mixed Methods, Realist Inquiry, Simulation Training, Triage, Virtual Reality

## Abstract

**Background:**

Because health professionals can end up being first responders to a mass casualty incident, they must train to improve preparedness and increase the preconditions of victim outcomes. Training and learning on how to handle a mass casualty incident is traditionally based on reading, lectures, training through computer-based scenarios, or sometimes through live simulations. Professionals should practice in realistic environments to narrow the theory–practice gap, and the possibility of repeating the training is important for learning. Virtual reality is a promising tool for realistic and repeatable simulation training, but it needs further evaluation. This study aimed to describe the acceptability and applicability of using VR for training in mass casualty incidents.

**Methods:**

A mixed-methods evaluation design was used, where the qualitative and quantitative findings were embedded into the discussion with a realist inquiry approach. A virtual reality simulation with mass casualty incident scenarios, named GoSaveThem (www.crash.nu), was used, and the participants were directed to perform triage. After the simulation, the participants filled in a questionnaire with open-ended questions and ratings on technical aspects, learning experiences, and improvement of preparedness. Eleven of the participants underwent interviews. The qualitative data was analyzed either summarily or with a conventional content analysis. Data were extracted from computer recordings of how long it took for each participant to triage the first 10 victims and to what extent the triage for the first 10 victims was correct. Descriptive statistical analyses were done, and a comparison was made to see if there were any differences between age, sex, educational background, and previous experiences that affected the outcome of triaging.

**Results:**

Training with virtual reality enables repeatable and realistic simulation training of mass casualty incidents. The participants expressed motivation to repeat the training and experience expanded virtual reality scenarios. This study shows that the acceptability and applicability of using VR for training MCIs were high overall in all examined dimensions for most users, with some exceptions.

**Supplementary Information:**

The online version contains supplementary material available at 10.1186/s12909-025-07319-z.

## Introduction

Preparedness for mass casualty incidents (MCIs) has become more important among healthcare professionals following disasters, conflicts, and terrorist threats worldwide. Failure to respond appropriately to an MCI may have adverse consequences for the victims [1, 2]. The primary objective of any MCI is to obtain the best possible outcome for the greatest number of individuals affected [3]. A heavy workload often strikes healthcare professionals who are first responders to an MCI, and they may not be experienced in managing these high-demand events as MCIs are still low-frequent in most parts of the world [4]. The work tasks in an MCI include assessing and treating victims, problem-solving, acting on safety and security matters, and overall decision-making on allocating available resources [5]. To improve preparedness and victim outcomes, healthcare professionals who can become first responders must train in MCI response and work tasks [3]. This study assumes that Virtual Reality (VR) can be an effective tool for training and learning about mass casualty incidents (MCI). However, it needs to be accepted and applicable to the users, and by using a mixed-method design with a realist inquiry approach, qualitative and quantitative data will be used to test this assumption and gain a deeper understanding of VR’s potential in this context.

## Background

Traditionally, healthcare professionals train and learn their skills to manage and prepare for MCIs using methods such as reading, lectures, playing table games, or using computer-based scenarios with virtual patients [6] and, occasionally, live simulation training with simulated patients [7]. It can benefit professionals to engage in training within realistic environments, and the ability to repeat the training is important for learning [8]. Reading and lectures risk leading to a theory–practice gap, meaning the learner might not use the obtained theoretical knowledge in practice [9]. Live simulations with simulated patients [10] require a lot of resources and are challenging to repeat [11]. When training for MCIs, high-fidelity virtual reality (VR) emerges as a tool for facilitating realistic and repeatable simulation training; however, VR necessitates further evaluation to be implemented wisely [7].

Different types of simulation training with VR have been developed simultaneously in several countries. The definition of VR is broad, making evaluating the concept of VR challenging [7]. The rapid advancement of artificial intelligence (AI) and the growing interest in technological solutions for alternative educational methodologies are likely to enhance the utilization of VR in training environments even more [12]. However, more than sophisticated VR technology is needed for training and learning. VR training should incorporate a rigorously designed curriculum aligned with its educational objectives to ensure favorable learning outcomes [13, 14]. Conversely, if VR training is inadequately planned, constructed, and implemented, learners may find themselves unable to learn, regardless of the sophistication and immersion of the VR technology [15].

An important feature of using VR for training and learning MCIs is its capacity to record and review scenarios, facilitate self-reflection and correction, and allow the health professional to repeat until learning occurs [7, 16, 17]. This capability encourages active engagement in the learning process, allowing the VR users to construct their understanding rather than merely absorbing pre-existing knowledge [8, 18]. Such an approach supports transitioning from traditional instruction to a more user-centered educational model [15]. However, to successfully implement new educational tools, the tools should be user-friendly and safe, and to achieve this, it is important to understand how applicable they are and how well the users accept them [7, 19].

Critical realism is a theoretical framework that can help explain the applicability and acceptability of educational interventions such as VR [20]. The strength of critical realism is its potential to explain how and why interventions work or fail to work in different contexts and for different people [21, 22]. A pragmatic methodology for research based on critical realism is a realist inquiry approach. Realist inquiry concerns what works for whom, under what conditions, how, and why by focusing on relationships between contexts, mechanisms, and outcomes. In this case, the context includes fixed settings like the VR equipment and technical features, as well as variable human factors like cultures and trained behaviors. Mechanisms operate at a psychological level to explain how and why people respond to interventions within specific contexts. According to realist inquiry, outcomes are the intended and unintended impacts of the intervention [21]. Therefore, critical realism was adapted as this study's theoretical approach, and realist inquiry was used to carry it out, primarily out of the assumption that the research question can best be understood by investigating multiple aspects. This framework can help explain how and why VR works in the context of MCI training.

How successful VR will be in the future for training MCIs depends on the development and functionality of the VR setup. Hence, developers and educators must know how to use and optimize VR as a pedagogical tool for training MCIs. Our assumption is that VR can be accepted and applicable for MCI training, but this needs further evidence. Therefore, this study aimed to describe the acceptability and applicability of using VR for training in mass casualty incidents.

## Methods

### Design

**Fig. 1:**
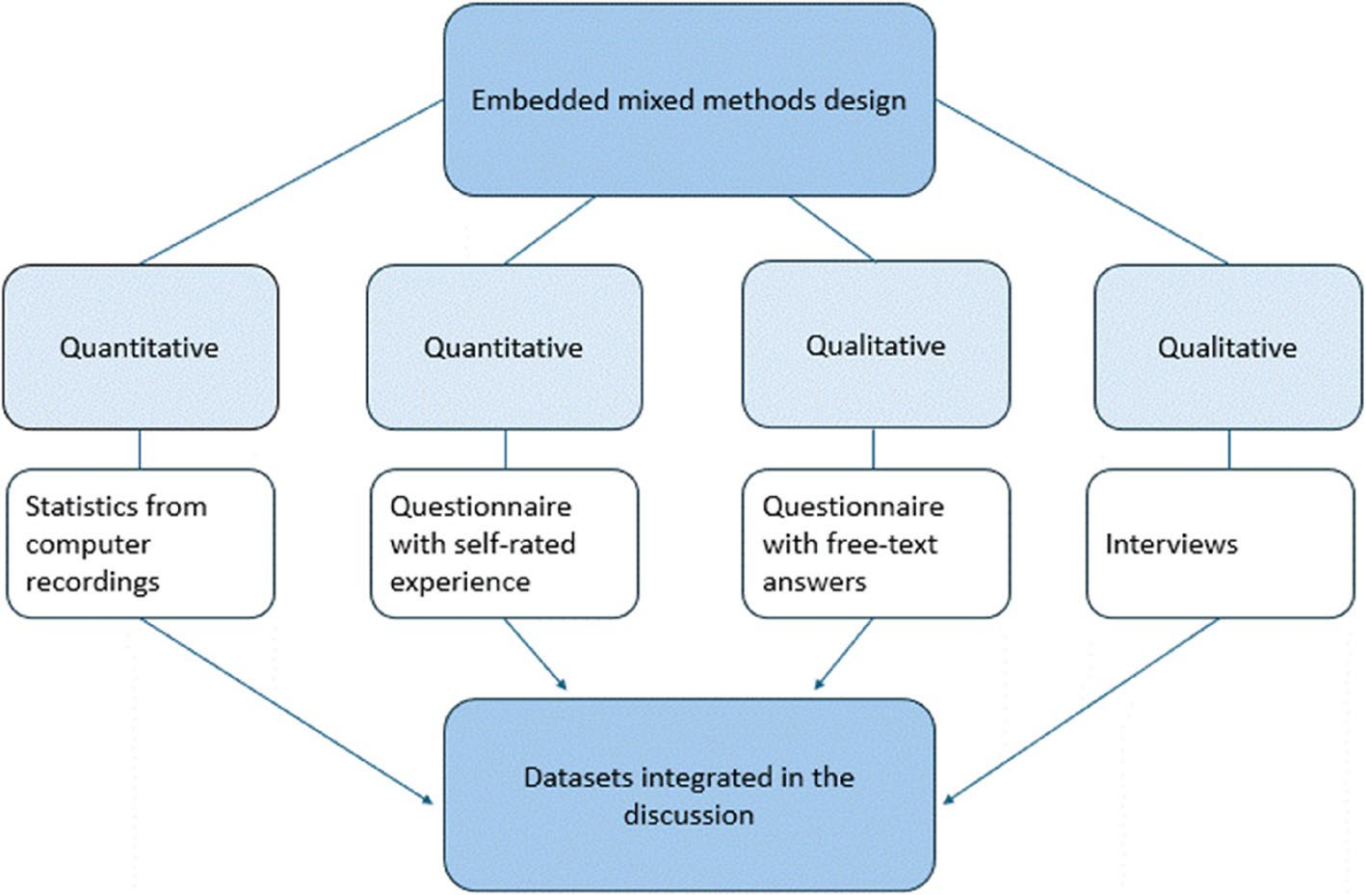
Embedded mixed methods design

To answer the study's aim, multiple data collection sources were used and analyzed to provide an expanded understanding of the acceptability and applicability of using VR for MCI training. A mixed-methods evaluation design was used [23], where the qualitative and quantitative findings were embedded into the section of discussion with a realist inquiry approach. Different quantitative and qualitative data were collected to answer the complementary study aim (Fig. 1). The study was conducted in line with the standards of mixed methods reporting guidelines [24].

### Participants

Convenience sampling strategies were used. Informational posters and emails were distributed to invite students from two universities and one ambulance service contracted by the county Council, to which two of the authors are connected as lecturers and nurses. In total, 95 participants were included (Table 1), of which 17 were specialist ambulance nurses, 35 were registered nurses, 37 were nursing students, and six were Ambulance clinicians (EMT). The ages of the participants ranged between 21 and 62 years, and the majority (65%) were female. Eleven of the participants were asked to participate in the interviews (Fig. 1). Those participants were asked as they were conveniently reached. They were employed in special ambulance units on standby for a substantial time during their working hours and were, therefore, available for VR training and interviews on duty. To work in a special ambulance unit, one is supposed to be senior and have theoretical and practical education to lead the work at major incidents and is supposed to help other ambulances with structure and organization in MCI situations. Their length of experience in the profession ranged between 6 and 18 years (m = 10,1/md = 9), and all of them had clinical working experience before getting employed. The ages of the participants ranged between 33 and 56 years (m = 41.2/md = 40), and the majority were male (*n* = 7).

**Table 1:** Descriptions of participants

(*n* = 95)		n =	%	Range	Median	Mean	Std.D	Missing *n* =
Gender male/female		33/62	33/35					
Age				21–62	32	33.8	8.6	6
Specialist nurse		17	18					
Registred nurse		35	37					
Nursing student		37	39					
EMT		6	6					
Years of experience in profession				1–33	2	4.6	5.3	
Experience of primary triage	Yes/No	47/48	49/51					
Experience of using VR	Yes/No	23/72	24/76					

### Setting and scenario

Data were collected in two cities in Sweden, Stockholm and Umea, from March 2021 to April 2024. A VR software with MCI scenarios, GoSaveThem®[25], was used. This software was selected because it was the only known available application adjusted to Swedish conditions, with the intention of making the setup as true to the local environment for the participants as possible. In the VR simulation, the participants were directed to perform primary triage, a system to initially sort out whom to help first, intending to save as many victims as possible [26–28]. The VR training was performed with a fully immersive head-mounted display from HTC VIVE®, enabling the participants to walk around in the virtual world and interact with the avatars. Handheld controllers with vibrations allowed participants to simulate assessing a real patient's pulse in the VR scenario. Breathing sounds played when participants brought their heads close to the avatar's chest, enabling realistic respiratory rate assessments. The controllers also enabled actions like applying tourniquets, using a torch, checking a watch, viewing triage guidelines, and assigning triage colors.

At the start, the participants received verbal information about the scenario, and then they familiarised themselves with the VR technique by testing an MCI scenario not included in the data collection. The participants got the chance to ask questions and said when they felt ready to start the research scenario. These pre-data collection preparations to familiarize with the VR technique generally took 5–15 min. The VR training was performed individually, with one researcher or assistant researcher nearby ready to reach out if the participant had questions or problems with the VR technique.

When starting the research VR scenario, the participants found themselves outside an ambulance on the street next to a subway station entrance. The scene that faced them was a chaotic site after an explosion in a subway station. Down the stairs, there was a train standing by the platform and a lot of bricks and dust on the ground. Victims (avatars n = 22), crying and screaming for help, were lying everywhere. In the farthest train carriage, the light was out, but while searching through the carriage, the participants, if they looked around, could find an open bag with unexploded explosives. The VR scenario ended when the participant decided to evacuate after finding the explosives, alternatively after all the victims were assessed and received their primary triage, or when the researcher decided the time was out after approximately 20 min. All participants assessed and triaged at least 10 victims before the VR scenario ended.

### Data collection

Directly after the VR scenario, all participants (n = 95) were asked to fill in a questionnaire with demographic questions and ratings (appendix 1) on how they experienced the VR training regarding technical aspects, usability, learning experiences, and improvement of preparedness, graded on a seven-point Likert scale. The questionnaire also included four open-ended questions.

Eleven participants also agreed to undertake individual interviews. These interviews had one open question,*” Can you describe your thoughts on the training with VR?”* and follow-up questions based on their answers, such as *“Can you give any example?”, “describe more”* and,*”in what way?”.* The interviews were audio-recorded and transcribed verbatim.

Furthermore, data were extracted from computer recordings of all participants (n = 95) on how long it took for each participant to triage the first 10 victims and to what extent the triage for the first 10 victims was correct.

### Analysis of the data

The analyses of the statistics were explorative and included a series of statistical tests depending on the type of variable, JASP version 0.19.1 was used. The descriptive data was explored numerically, visually, and statistically with a Shapiro–Wilk test and found not to be normally distributed. Therefore, the non-parametric Kruskal Wallis and Mann–Whitney U test was used to explore differences between the groups in statistical data. A *p*-value of < 0.05 was considered statistical significance. In assessing effect sizes, rank biserial (rB) correlation was used to categorize as small (0.1), medium (0.3), or large (0.5).

The qualitative data were analyzed in two different ways. First, the first three open-ended questions of the questionnaire were analyzed with a summative content analysis [29], as those questions ended up in short answers with related meanings. Searches for occurrences of related words were grouped and then counted.

Secondly, the fourth and last question of the questionnaire, “Do you have other comments?” had richer content and was analyzed using conventional content analysis [29]. As a similar question was asked in the eleven interviews, it was decided to analyze these qualitative data altogether. The interview transcripts were read and re-read, and the meanings were condensed. All condensed meaning units and written answers from the last question in the questionnaire were coded. Codes were then grouped into sub-categories and categories through discussions by two researchers (SH, VL) until a consensus was reached. The final categories were decided through research group consensus.

The model of mixing the collected data was through integration, which occurred during the discussion of the results [23] with a realist inquiry approach [21].

## Results

The results below are presented in four different parts. First, the results of the self-rated experience of using VR are presented. Then, the results from three questions of the questionnaire, in which the participants described their experiences of VR, are summative presented. Thirdly, the last question of the questionnaire is presented together with the results of the interviews regarding other comments on using VR. Lastly, the results of the data extracted from the computer are presented regarding how quickly and correctly the participants acted during the VR simulation.

## Self-rated experience of using VR

The questionnaire had six questions, answered on a 7-point Likert scale, where 1 was very low and 7 was very high. The questions aimed to evaluate the participants’ experience of the VR training (Table 2). The results were not normally distributed and are presented below by range, median, mean, and standard deviation. The results showed that participants rated their experience highly on average.

**Table 2:** Experiences of using VR

Question	Range	Median	Mean	Std. D	Missing *n* =
1. To what extent do you feel that the training in VR resembles reality?	3–7	5	5.4	1.1	2
2. To what extent do you think that VR training can prepare you for similar situations?	4–7	6	6.2	0.8	2
3. To what extent did you find the technology and equipment easy to handle?	2–7	6	5.8	1.2	2
4. To what extent was the information you received before the exercise sufficient to complete it?	2–7	7	6.5	0.9	2
5. To what extent do you feel that VR can be a tool to evaluate and improve your performance?	3–7	7	6.4	0.7	2
6. Training with VR will help me in the care of patients with similar conditions	4–7	6	6.1	1.0	2

## Summative content analyses of the questionnaire

### Question 1: Describe if and in what way you experience any physical symptoms in connection with the use of VR

**Fig. 2:**
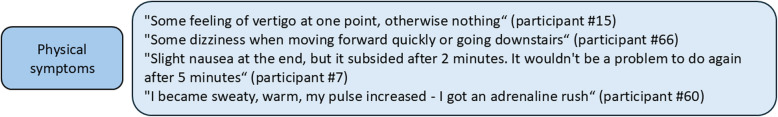
Examples of quotes from the summative content analyses

A majority of the participants expressed some dizziness during or after the VR training (61%). Most of them described the dizziness as mild and manageable (37%). The dizziness was worsened by rapid movements and changes in level, such as walking downstairs in the scenario. Mild and quickly transient nausea was another physical symptom that participants described (28%). Some of those who became nauseous had more nausea at the beginning (4%), and some became a little nauseous towards the end of the exercise (3%). There were also participants expressing feeling nauseous without putting the word"a little"before, which could be interpreted as the nausea being more bothersome (20%). For two (2%) of those participants, the nausea became so severe that it affected the quality of the training. Some participants experienced becoming sweaty and hot during the training (10%), and three (3%) of them also described this in connection to having an adrenaline rush. Three (3%) participants stated they had no physical symptoms, and four (4%) did not answer the question (Fig. 2).

### Question 2: In summary, how satisfied are you with practicing with the help of VR?

**Fig. 3:**
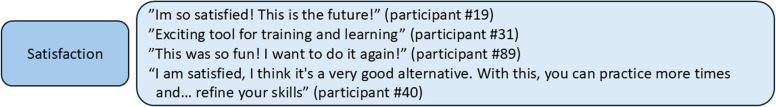
Examples of quotes from the summative content analyses

Most participants (90%) were either satisfied or very satisfied with the VR training of MCIs. The majority (65%) of the participants expressed in different ways that they were thrilled, wanted more VR training and believed this could be the future. Two participants (2%) expressed that they were quite satisfied or not sure if they were satisfied. Seven participants (7%) did not answer this question (Fig. 3).

### Question 3: Was the allocated time sufficient to complete the VR training satisfactorily?

**Fig. 4:**
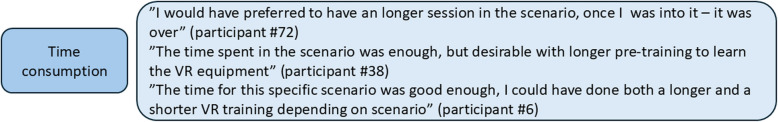
Examples of quotes from the summative content analyses

Most participants felt that the time they spent in the VR training was optimal (72%). Some participants expressed that they had wanted to practice more (20%), either for a longer time or repeatedly. Some (9%) indicated they wanted to practice more because it was fun, but others thought they wanted to learn the equipment better and, therefore, needed more time (5%). Two participants (2%) wrote that the VR training was too long and would prefer several shorter practices rather than one long practice. Two participants (2%) had to stop the exercise; they did not give a clear answer to the question. Four participants (4%) did not answer the question (Fig. 4).

## Conventional content analyses of “other comments” questions

**Fig. 5:**
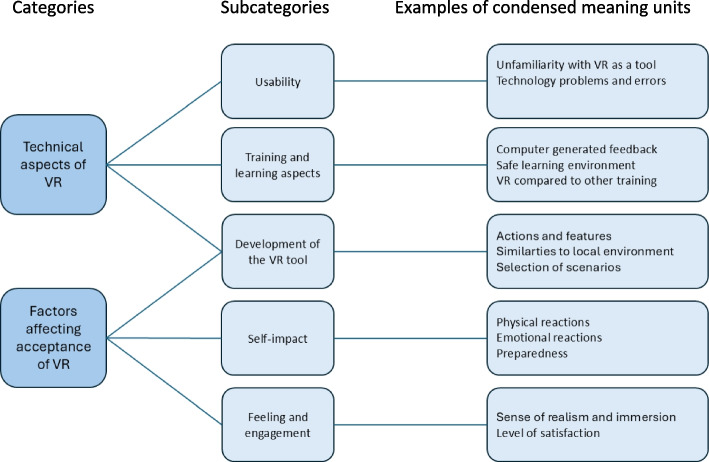
Categories and subcategories of answers to “Do you have other comments?”

The fourth and last question in the questionnaire was, *“Do you have other comments?”* The starting question in the eleven interviews was” *Can you describe your thoughts on the training with VR?”*. Using conventional content analysis, two categories and five subcategories were created out of the answers (Fig. 5).

The subcategories are presented in more detail below.

### Usability

The participants described reasons for taking a long time to proceed with assessing and triaging the victims during the VR scenario as usability problems. One reason described was a lack of experience playing computer games and unfamiliarity with handheld controllers. For those not identifying as “gamers”, it was described as extra important for the VR technology to be user-friendly. These participants believed it took longer time to complete assessments and act because of uncertainty and confusion about which button to use, which was described as reasons for a prolonged time to walk and move around the environment than it would have in real life. Usability issues were the most described reason for the loss of time during the VR scenarios.

*“It had gone faster in real life, so in the beginning, it took longer to find the right triage color” (participant #4)*

Participants were also describing technical problems with the sound and having problems getting sharp vision in the goggles. The experienced technical problems were suggested to be remedied to improve usability. Slowing down the joystick's speed in the handheld controllers was thought to make walking easier. The experienced sound and picture problems were disturbing, and there were comments that the VR technique should work even for those with different kinds of physical obstacles, before being implemented widely in educational contexts.

*“That it works for people with glasses is a prerequisite so that it can include as many people as possible” (participant #76)*

### Training and learning aspects

There were different opinions on training and learning aspects from a technical point of view after the VR scenario. The participants described the possibility of getting computer-generated feedback and how this may open up the possibility of self-practicing their skills. The participants expressed a desire for easy access to VR for repeatable training with feedback that can be used when needed, not only when a human instructor is available. Furthermore, the participants said they felt safe if they made mistakes during VR simulation for several reasons. No one can get hurt, which makes the performance anxiety decrease.

*“So here we have the opportunity to fail, this gives us the tools to… work on our skills” (participant #14)*

The participants also compared VR to other simulation training methods in training and learning for MCIs. They expressed enthusiasm for VR regarding the ability to repeat the training and improve the learning opportunity, compared to live simulations. MCI live simulations were described as expensive, carried out too rarely, and messier. In live simulations, the participants described that more people are involved, and there is less focus on individual skills. However, as a negative aspect of VR, the participants described less empathy in the VR scenario than in live simulation training with other humans and less realism when performing some hands-on care.

*“But, we don't get the feeling of putting a tracheal tube down, no feeling of creating a free airway, or putting an avatar in a prone position, you have to do that hands-on.” (participant #3)*

### Development of the VR tool

The participants had several development suggestions for improving VR training. Suggested improvements were the ability to include more disturbances and challenges in the scenarios, to make it more like real-life MCIs, such as more involved people and possibilities to interact. More sense impressions, such as smell, sound, and vision, could make a big difference.

### “You don't smell it, nor does it create the stress feeling it could have” (participant #3)

The ability to communicate with others, face-to-face or via radio communication, would be an advantage for the VR training, as communication in MCIs was considered challenging and improvable. The participants also suggested an ability to provide more caring actions for the victims (avatars). Moreover, the participants commented that the more the virtual world looks like reality, the more it elevates the ability to enter the scenario in an immersive way. When reminded that it was a computer game because something looked unrealistic or not like the local environment, it made them feel less immersed.

*“I sometimes get disturbed about the environment; it should really look like... I understand that maybe it's not... it's not created... but the ambulance was still similar to the one we use. So little is inspired anyway, I guess. But the police officer looked like American police, not Swedish.” (participant #18)*

The participants also wished for a wider range of scenarios to enable variation and different kinds of challenges. They had many ideas about what you can develop with more scenarios, from regular patient visits to deviation report cases in a VR environment, where your post-VR training can go through what happened and how to improve. Further, the development of VR was also described as affecting the level of acceptance of the tool. The more development, the better acceptance.

*“I like the concept, but more disruptions and variance could be good and make it more useful and realistic…and accepted” (participant #45)*

### Self-impact

The participants explained how factors making an impact on themselves were affecting their acceptance of using VR in different ways. One of the self-impacting factors was experiencing physical reactions during the VR training. There were elaborations on whether a predisposition for motion sickness creates more trouble. They believed that nausea could be the biggest obstacle to a wider adoption of VR training if it is not resolved. Emotional reactions during the VR training were expressed as not as strong as they would have been in a real-life MCI, especially with children as victims in the VR scenario. It was also described as being emotionally less difficult to make mistakes than in reality.

*“No, this is still easier to dismiss emotionally. On the other hand, it feels like I'm entering my mode here, entering my work role” (participants #3)*

The participants described the reason for training in MCI scenarios and how it helped them become better prepared for real-life MCIs. They also highlighted insights about themselves regarding their lack of skills, both regarding how to triage during an MCI and how to handle safety and security issues during a suspected terrorist attack.

*“I have discovered a lot of things that I have done wrong as well, that I would have liked to do differently” (participant #10)*

The possibility of reviewing one's actions after the VR training and doing reflections was described as a benefit of using VR. Most behaviors reflected upon were insights about unexpected tunnel vision and how that affected their actions and movements in the VR scenario. Reflections on their decision-making during the scenario showed that participants were satisfied with themselves, making decisions they had learned, and thereby living up to their expectations of self. There were also reflections on how some decisions were found easier to make in the VR scenario than supposed in real life.

*“It was easier in the game than I thought to set the triage color black [dead]” (participant #7)*

### Feeling and engagement

The participants also expressed feelings about the VR training, which was not directly associated with previous subcategories. There were several different feelings and aspects of how they enjoyed and engaged in the VR training. The level of realism was described as crucial for empathy and engagement. Satisfaction with the VR training was the predominant and most common comment. They motivated why they were satisfied with this type of training by being fun, exciting, rewarding, and informative.

*“It was very, very good… because we practice so little, and try to familiarize ourselves with how it might be in reality, it felt quite realistic, I think” (user #16)*

## Data extracted from computer recordings of the VR training

Results from computer recordings (*n* = 95) show that the number of correct triages of the first 10 victims ranged between 4–10 (md = 8). The time it took for the participants to triage the first 10 victims was 3.10–15.57 min (md = 7).

**Fig. 6:**
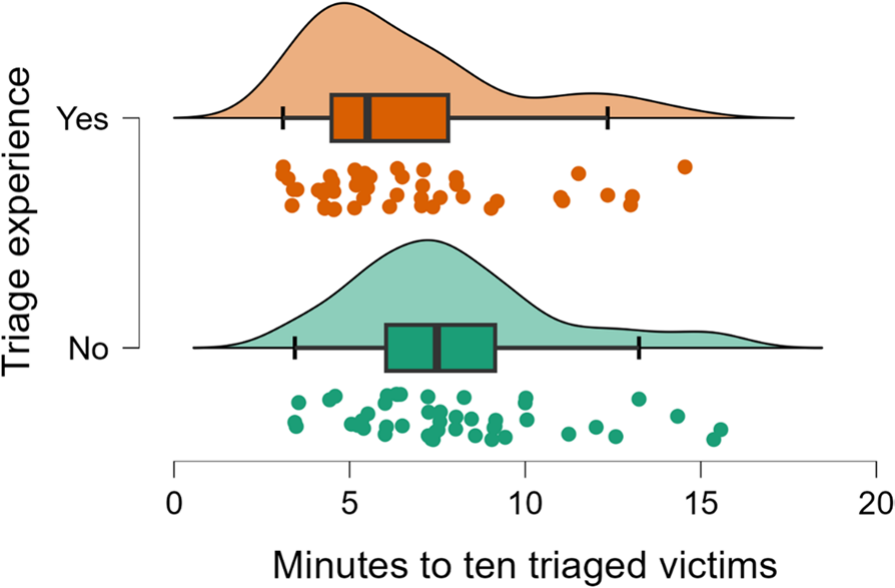
Number of minutes for triage of the first 10 victims

**Fig. 7:**
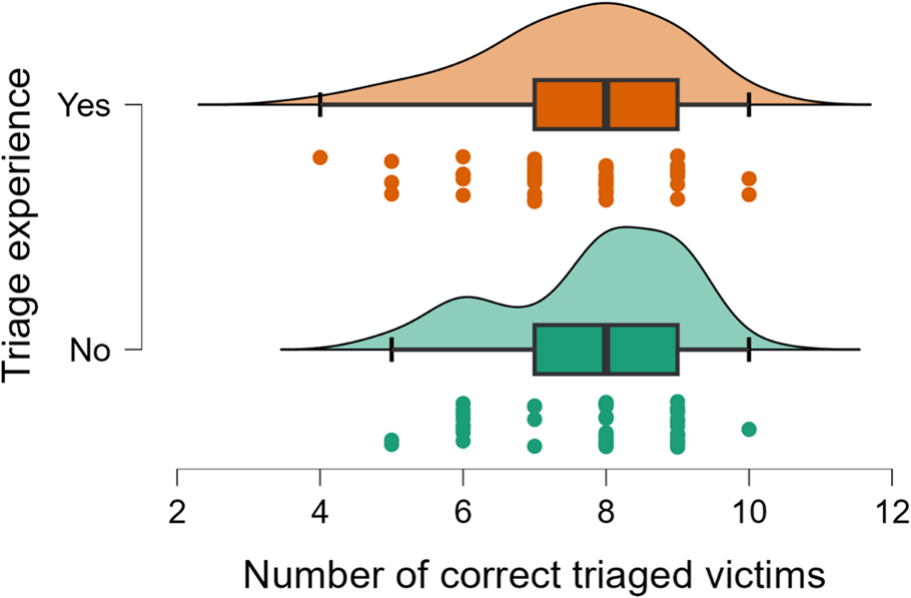
Correct triaged victims, out of 10

Different types of statistical tests were done depending on the type of variable, but no significant difference was found between different ages, genders, educational backgrounds, and previous experience of using VR that affected the outcome of triaging timely and correctly. However, a Mann–Whitney U test showed a statistical difference (*p* = 0.005) between those with prior experience in performing triage and those with non-prior triage experience on how many minutes it took to triage ten victims (Fig. 6). The rB = 0.336 suggests this is a medium effect size. However, no significant differences were found regarding the number of correct triaged victims between the groups (Fig. 7).

## Discussion

The main finding in this study was that the acceptability and applicability of using VR for training for MCIs seem to be high in all examined aspects for most participants with some exceptions, which confirms our study assumption. This means that VR training for MCIs seems to work very well for the majority. Still, there are outliers, and the acceptability and applicability are lower for those who experience obstacles of different kinds. Overall, there was consistency and consensus in the quantitative and qualitative data. In line with the theoretical framework, critical realism, the acceptability and applicability of using VR for training MCIs are discussed below from the realist inquiry perspectives of context, mechanisms, and outcomes [21].

### Context

The context, described in realist inquiry, highlights fixed settings such as technical features, and variable human factors like cultural aspects and trained behaviors [21]. In this study, the VR tool and technical features had high ratings regarding the extent to which the users felt that the training in VR resembled reality. This finding was supported by the qualitative answers regarding how the experience of immersion and reality in VR training was important to training behaviors for real-life MCIs. However, the results suggest that greater sense impressions, such as smell, sound, and vision, could have the chance to make a big difference in context realism if added as features in future VR training. Furthermore, the VR training was suggested to be as true to the local and cultural context as possible. Overall, the participants found the technology and equipment easy to handle, but some expressed technical issues as obstacles to immersion and usability. The level of realism, as the appearance of disruptions and variance, seemed to be crucial for VR's acceptability, a result supported by other research [7, 9, 12, 15, 16]. The most commonly expressed answer to the question “Do you have any other comments?” was different development suggestions for the context. The acceptability and applicability of VR would probably be even more rigorous when more actions and features are accessible to improve the context and if some of the technical problems found in this study are erased.

### Mechanisms

The mechanisms, described through realist inquiry, explain at a psychological level how and why people respond to interventions within specific contexts [21]. One of the key findings in this study was the expressed satisfaction and enthusiasm for VR as a tool to train MCIs, both in ranking numbers and as an answer to directed and open questions. Psychological aspects, such as positive attitudes toward VR as a pedagogical tool, can speed up the implementation of VR [12] and potentially affect the level of acceptance in education. This result is supported by other research, where it is found that VR used as a pedagogical tool effectively enforces greater enthusiasm amongst the participants [30–34], and has the potential to enhance education by improving both theoretical and practical knowledge as well as satisfaction [30, 35, 36]. Being satisfied and experiencing enjoyment when training and learning can be motivators for attending classes and learning new skills, and it encourages concentration and helps absorb knowledge [37].

The participants rated their belief that training with VR could improve their performance and preparedness for similar situations and that getting better preparedness for real-life MCIs is a key element of MCI training [1–3]. The self-impact of the VR training was described emotionally and physically in both the quantitative and qualitative results. Minor physical reactions were common, but some participants described them as so bothersome they couldn’t perform the training, negatively affecting both the acceptability and applicability of VR. How to deal with and erase physical symptoms created by VR training could be a subject for further research. The physical reactions were surprisingly common (61%), and the reason being connected to this specific VR setup cannot be ruled out. The ones who had no previous experience using VR tended to have more physical reactions, which could be interpreted as being less likely to get physical symptoms when getting used to VR, but these numbers are not statistically significant. However, the high level of physical reactions could also be due to the questions specifically asked regarding the matter in this study.

### Outcomes

The outcomes in a realist inquiry are the intended and unintended impacts of the intervention [21], which regarding the VR training were described from different angles. The participants rated the training with VR highly, believing that the training would help care for patients with similar conditions in the future. The participants highlighted the positive aspects of learning as an important outcome of VR training, making it applicable to training and learning how to handle an MCI. As described in other research, VR offers a safe learning environment [7, 32, 38, 39], allowing learners to make mistakes without jeopardizing patient safety and outcome or exposing themselves to danger during training. This study confirms this advantage of VR, and the participants described nuances of why training with VR felt like a safe environment for them. This feeling of safety can foster more audacious decision-making and may facilitate future exploration of new standards.

This study shows no significant differences found between age, gender, educational background, and previous experience of using VR that affected the results, which was surprising. It was expected that younger participants, who had previous experience using VR, would act quicker and more correctly when performing triage so as not to be disturbed by learning new technology and able to concentrate only on the triage task. However, this should be interpreted cautiously, as confounders may bias the data, and the number of participants in this study is relatively small. Reports show that learners who have grown up with the Internet and are used to playing video games may have a more intuitive capability of using VR [15, 19, 40, 41], and young healthcare professionals can therefore be expected to be different from previous generations. However, if the data from this study is correct, it can be interpreted that VR works well for most people, regardless of age or previous experience of using VR. If so, educators would be challenged to develop reliable and valid methods for VR, which are pertinent to the educational programs in the short and long term, and consider expanding VR training to more learners [40].

The only significant difference between groups (*p* = 0.005, rB = 0.336) was how many minutes it took for the participants to triage the first 10 victims, where those with previous triage experience were faster than those with non-prior triage experience. If this difference is persistent in other educational settings, it is a subject for further research. However, this result aligns with the conviction that one gets more effective at tasks after repetitive training [8, 11].

Further research is needed to ascertain VR’s suitability for training MCIs and compare it to other pedagogical tools. Ultimately, VR can be a viable research instrument for examining training protocols for MCIs and a platform for developing preparedness, assessing learning outcomes, knowledge retention, and performance accuracy.

### Methodological considerations

The findings in this study are limited in that they reflect the acceptability and applicability of using VR for MCI training amongst participants in Sweden. However, participants from the northern parts of the county and the capital in the southern parts were included, and their environments differ in many ways. Moreover, only one VR application is used, with its limitations and strengths, and caution should be taken before generalizing the results to other VR platforms. Especially technical and contextual conclusions from this study are related to this specific VR system and setup. However, an assumption is that the knowledge from this study may be transferable to some extent to comparable contexts.

It could be questioned whether triage is a stable variable to use to explore the acceptability and applicability of VR for training MCIs. Users tend to perform both under- and over-estimations with the triage tool [42]. However, triage is a well-known task to perform during MCIs worldwide [28], and training on that task in a VR setting can be a realistic way of exploring the acceptability and applicability of VR as a tool for MCI training.

The first, third, and last authors had contextual pre-understanding before conducting this study, as they have a background as health professionals being trained as first responders to MCIs. This may have influenced the understanding of the data, as well as helped them understand the nuances when analysing and interpreting results. The authors aimed to maintain a critical approach and a journal with field notes was written during the research process to keep notes of reflection on the process and the researchers’ role and influence [43, 44]. To avoid possible pre-understanding bias problems, the second author reviewed the data and results iteratively [44]. The use of a mixed methods methodology can be seen as a strength for the validity and legitimacy of the results in this study, as it allowed both to explore statistics as well as provide an in-depth understanding of the participant’s actions, perceptions, and experiences to understand further the acceptability and applicability of using VR for MCI training.

## Conclusions

Healthcare professionals who can end up being first responders to an MCI should train to improve preparedness and increase the preconditions of victim outcomes. VR is a pedagogical tool for facilitating realistic and repeatable simulation training. This study shows that the acceptability and applicability of using VR for training MCIs were highly rated overall in all examined dimensions for most users, with some exceptions, such as experiences of technical disruptions and physical symptoms. The continuous development and enhancement of VR technology as a pedagogical tool for MCI training warrants further investigation and can potentially fill an educational gap in MCI preparedness.

## Supplementary Information

Supplementary Material 1.

## Data Availability

The datasets used and analysed during the current study are available from the corresponding author on reasonable request.

## Abbreviations

MCI: Mass Casualty Incident
VR: Virtual Reality
EMT: Emergency Medical Technicians
