# Supplementary material for: Acceptability and applicability of using virtual reality for training mass casualty incidents- a mixed method study
Source: BMC Med Educ. 2025 May 19;25:728. doi: 10.1186/s12909-025-07319-z (PMC12090617; doi:10.1186/s12909-025-07319-z)
Supplement: Supplementary file 1 — Supplementary Material 1. [file 12909_2025_7319_MOESM1_ESM.pdf]

1. To what extent do you find the VR training is similar to real life MCIs?

Very low ○ ○ ○ ○ ○ ○ ○ Very high

2. To what extent do you believe that training with VR can prepare you for similar situations in reality?

Very low ○ ○ ○ ○ ○ ○ ○ Very high

3. To what extent did you experience the technique and equipment were easy to use?

Very low ○ ○ ○ ○ ○ ○ ○ Very high

4. To what extent did you experience that the VR technique can be a support for evaluating and improving your performance?

Very low ○ ○ ○ ○ ○ ○ ○ Very high

5. To what extent do you believe that training with VR will help me when caring for patients in similar situations in real life

Very low ○ ○ ○ ○ ○ ○ ○ Very high

Appendix 1
